# School Attendance and Health

**Published:** 1917-11-24

**Authors:** 


### SCHOOL ATTENDANCE AND HEALTH.

BY A SOCIAL WORKER.

THE announcement of the dropping "for this session" of Mr. Fisher's Education Bill is received with mixed feelings not merely by the public but by individuals. On the one hand, it proposed embarkation on an uncharted—or, rather, very variously charted—ocean, whose tides and undercurrents could only be guessed by certain ominous ripples on the surface. On the other, those who see the conditions of the children's lives cherished hopes of seeing the end of certain tragic things.

"They talk of saving the kiddies" said, a few days ago, a woman whose "war work" lies among them, "but I tell you they're not being saved! When I come down this street at seven on these cold mornings I see the mothers who are going out to work taking their children to the 'minders.' One will have a child tucked under each arm, sobbing perhaps, or actually asleep. Another is pushing a pram, and it's raining and the child getting soaked, and she drags another by the hand faster than its legs can go. The bit of bread it carries is getting wet too, it drops, the mother picks it from the mud and gives it back and shakes the child. No wash, no hot drink before they come out, no change of shoes or clothes, and only the bread to eat and the minder-woman's stuffy room to play in. She'll not cook for them, or dry them, or wash them either, and what will the mother do for them when she comes back after a day's work to take them home?" There will also be older children at play in the cold, dark streets, or in mischief there, during winter evenings.

*The problem of school attendance is, in the main, a medical problem*, writes Sir George Newman in his Report to the Board of Education (the italics are his own). Elsewhere he points out that the School Medical Service and the Education Authority cannot be wholly responsible. "Before the child comes to them it is often marked or maimed by its previous experiences; when it leaves them it often passes under conditions which modify, impair, or even destroy the good effect produced by their measures. . . . They are partners in the undertaking." Its very birth, in fact, may have permanently injured the child, and all through the nine years of its school life its home may be an agency that to some extent vitiates the effort of the educator. You cannot train and inform the mind which is being debilitated by the encasing body. On the other hand, compulsion put upon the brain to work for the mind's development may still further injure the body which is not rightly conditioned. Sir George Newman finds two forms of irreducible minimum as regards this needful provision of health. First, the exigencies of the war have so reduced the Medical Service that the lower aim of merely detecting and, if possible, curing the sick child must needs replace that of guarding the normal child. The ambulance below the cliff, in fact, must replace the fence on top. But all this time the other "irreducible minimum" has become more evident, and Dr. Newman finds it to consist of seven points: (1) Periodical examination for medical *and dental* defects; (2) that the ill-nourished child shall be rightly fed, and the unclean child cleansed; (3) that the sick and defective shall have skilled treatment; (4) fresh air or open air during lessons; (5) suitable physical exercise; (6) that paid work should only be done by school-children under approved conditions; (7) that all influences injurious to health and development in the school and its lessons shall be eliminated.

Now these seven points, and perhaps especially the last, show clearly enough the need for women's work and for a better knowledge of hygiene by a large number of women, in order that they may help the depleted Medical and Nursing Services, not as enrolled members but as valuable auxiliaries. And this not only during war-pressure, but afterwards, if the needed research work is to be done, for Sir George Newman states that no fewer than 350 inquiries had been set on foot by school medical officers before such work became impossible. Repeatedly in his Report we find indications of the great advantages that would accrue if teachers and care committee workers possessed sufficient knowledge to note early symptoms and remedy minor ailments and to advise mothers, and to carry out in school many routine matters of personal hygiene. Thus it is said that every school should have the benefit of a nurse's daily visit, and that one nurse might make a round of five or six schools. "If, however, the head-mistress possessed the necessary knowledge," such an official might be dispensed with. Such watchfulness would do what is urgently needed to prevent the spread of epidemics. The Report testifies to the improvement gained by increased observation of "contacts" and "carriers," and the additional work done by teachers, nurses, and care committee members in consequence of the depletion of doctors. It may be said in passing that of the 1,300 medical officers 103 are women, whose work lies in fifty-six areas, and that five areas employ seven women dentists. That excellent new institution, the School of Dental Mechanics for Women, in Wigmore Street, wisely facilitates for its workers attendance at lectures and study of dentistry, which will no doubt furnish some useful recruits. But the pressing matter is the assistance of competent women in remedying minor evils, and bringing to medical treatment the cases that need it. The evidence of the Report is, for instance, very remarkable as to the good results obtained by appointments of women attendance officers. The Borough of Taunton, which for the past five years has employed no men in this capacity, shows an increase in the average percentage of school attendances, less absence on grounds other than medical, and fewer prosecutions. Sir George Newman notes in passing that the woman officer's visit is often helpful to mothers who think it wise to keep an adolescent girl at home.

The presence of teachers at medical inspections has done much to interest and enlighten them, and especially to enlist their help in securing cleanliness. It is a shock to read that in *secondary schools* there is still a percentage of 4.1 verminous heads, a decrease of .9 per cent. since 1915. The influence of cleanliness upon health needs to be insisted on to many parents. The child with vermin or scabies does not sleep well and suffers in nutrition. Many school doctors have drawn attention to the insufficient sleep of a number of children whose parents will not put them to bed before they themselves go. They say that the Daylight Saving Act has tended to reduce the children's nights by an average of one hour. Children with verminous heads contaminate others, and so lower the general health, while the presence of uncleanly bodies pollutes the whole air of the schoolroom. Swimming baths are found to be one of

**SCHOOL ATTENDANCE AND HELP**—(*continued*).

the best remedies for this trouble. The children know they will not be allowed to enter the bath if not clean, and therefore insist on being washed or permitted to wash at home. Epidemics spread faster and are more severe among children whose health is lowered by uncleanliness. Incidental points in the Report show how many gains in detail emerge from knowledge gained by teachers. For instance, at Walsall the establishment of an Eye Clinic revealed a serious amount of trouble. One of the teachers instituted for sewing lessons thread of a greenish hue and dark green material with very satisfactory results. Careful observation showed that this plan was preferable to that of introducing very coarse needles, as these demand too much "driving power" from the child. As regards actually blind children it is interesting to note the recommendation of the Departmental Committee that wherever possible blind teachers should be employed for them at salaries equal to those of the sighted.

The acquisition, then, of some kind of working knowledge of the hygiene of child-life by the woman of to-day and to-morrow is greatly to be desired. Whether as official, as teacher, as care worker, as friendly visitor, or for her own potential motherhood every girl needs some degree of this knowledge. And those who believe that in better education lies the true path to a better social life will take action when they understand *that the problem of school attendance is in the main a medical problem.*

